# How Does the Severity of Injury Vary between Motorcycle and Automobile Accident Victims Who Sustain High-Grade Blunt Hepatic and/or Splenic Injuries? Results of a Retrospective Analysis

**DOI:** 10.3390/ijerph13070739

**Published:** 2016-07-21

**Authors:** Ting-Min Hsieh, Tsung-Cheng Tsai, Yueh-Wei Liu, Ching-Hua Hsieh

**Affiliations:** 1 Division of Trauma, Department of Surgery, Kaohsiung Chang Gung Memorial Hospital and Chang Gung University College of Medicine, 123 Ta-Pei Road, Niao-Song District, Kaohsiung 833, Taiwan; hs168hs168@gmail.com (T.-M.H.); anthony0612@adm.cgmh.org.tw (Y.-W.L.); 2 Department of Emergency, Kaohsiung Chang Gung Memorial Hospital and Chang Gung University College of Medicine, Kaohsiung 833, Taiwan; takk921@yahoo.com.tw

**Keywords:** blunt hepatic and/or splenic injuries (BHSI), motorcycle, car accident, trauma, injury severity score (ISS), organ injured score (OIS), length of stay (LOS)

## Abstract

*Background*: High-grade blunt hepatic and/or splenic injuries (BHSI) remain a great challenge for trauma surgeons. The main aim of this study was to investigate the characteristics, mortality rates, and outcomes of high-grade BHSI in motorcyclists and car occupants hospitalized for treatment of traumatic injuries in a Level I trauma center in southern Taiwan. *Methods*: High-grade BHSI are defined as grade III-VI blunt hepatic injuries and grade III-V blunt splenic injuries. This retrospective study reviewed the data of 101 motorcyclists and 32 car occupants who experienced a high-grade BHSI from 1 January 2011 to 31 December 2013. Two-sided Fisher’s exact or Pearson’s chi-square tests were used to compare categorical data, unpaired Student’s *t*-test was used to analyze normally distributed continuous data, and Mann–Whitney’s U test was used to compare non-normally distributed data. *Results*: In this study, the majority (76%, 101/133) of high-grade BHSI were due to motorcycle crashes. Car occupants had a significantly higher injury severity score (ISS; 26.8 ± 10.9 vs. 20.7 ± 10.4, respectively, *p* = 0.005) and organ injured score (OIS; 3.8 ± 1.0 vs. 3.4 ± 0.6, respectively, *p* = 0.033), as well as a significantly longer hospital length of stay (LOS; 21.2 days vs. 14.6 days, respectively, *p* = 0.038) than did motorcyclists. Car occupants with high-grade BHSI also had worse clinical presentations than their motorcyclist counterparts, including a significantly higher incidence of hypotension, hyperpnea, tube thoracostomy, blood transfusion >4 units, LOS in intensive care unit >5 days, and complications. However, there were no differences in the percentage of angiography or laparotomy performed or mortality rate between these two groups of patients. *Conclusions*: This study demonstrated that car occupants with high-grade BHSI were injured more severely, had a higher incidence of worse clinical presentation, had a longer hospital LOS, and had a higher incidence of complications than motorcyclists. The results also implied that specific attention should be paid to those car occupants with high-grade BHSI, whose critical condition should not be underestimated because of the concept that the patients within in a car are much safer.

## 1. Introduction

Blunt abdominal trauma accounts for 2.7% to 17% of emergency department (ED) visits owing to trauma [1,2,3] and may result in life-threatening hemorrhage [4]. The liver and spleen are the most commonly injured organs in this region. Blunt hepatic and splenic injuries have been reported to occur in 4% and 5.4% of blunt torso injuries, respectively [5]. Of all the common causes of blunt hepatic and/or splenic injuries (BHSI), traffic accidents, falls, occupational accidents, and assaults account for more than 80% of cases [6,7]. In addition, although the incidence of BHSI in motor vehicle accidents is relatively low (<6%), compared to the incidence of injuries to the extremities or head injuries (>50%) [1,2,8,9,10,11,12], initial evaluation and management of patients with high-grade BHSI are still formidable challenges for clinicians because concealed hemorrhage remains the second most common cause of death after trauma [4]. Traffic accidents usually result in remarkable damage to the drivers or riders. With the increasing motorization in recent decades, motorcycle and car accidents continue to be a major source of BHSI [8]. In developed countries, BHSI occurs more frequently in car occupants, but the situation is possibly different in Taiwan, where motorcyclists comprise a major portion of the trauma population. In fact, more than 50% of all trauma accidents involve motorcycles [13,14,15,16]. Previous studies have also shown that the mechanisms of injuries in motorcyclists with BHSI, as well as the characteristics of resulting injuries in both anatomical and physiological aspects, are different from those of car occupants, taking into account the vulnerability, lack of protection, and relatively high driving speed of the former [9,17]. Because the mechanism of traumatic injury in Taiwan is distinct from that in many Western countries, the main aim of this study was to investigate the characteristics, mortality rates, and outcomes of high-grade BHSI in motorcyclists and car occupants hospitalized for treatment of traumatic injuries in a Level I trauma center in southern Taiwan.

## 2. Methods

### 2.1. Ethics Statement

This study was pre-approved by the Institutional Review Board (IRB) of the Chang Gung Memorial Hospital (approval number 102-4441B). The need for informed consent was waived according to IRB regulations.

### 2.2. Study Design

**Figure 1:**
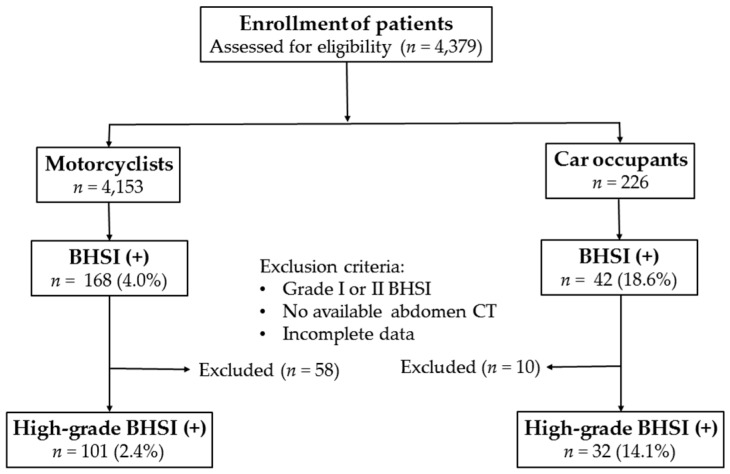
Flow chart of the studied patients with blunt hepatic and/or splenic injuries (BHSI).

This retrospective study reviewed all data added to the Trauma Registry System from 1 January 2011 to 31 December 2013 in a 2400-bed facility and Level I regional trauma center that provides care to trauma patients primarily from southern Taiwan. All data were prospectively collected and retrospectively analyzed from the medical records of the hospitalized patients with abdominal trauma due to motorcycle and car accidents. Only motorcyclists and car occupants were included, whereas pedestrians involved in the accidents and patients with BHSI from causes other than motorcycle and car accidents were excluded. The other injury mechanisms were excluded because there were too few patients with high-grade BHSI from all of the other injury mechanisms included fall, bicycle accidents, and strike against/by objects. The severity of BHSI was graded according to the classification of the American Association for the Surgery of Trauma (1994 revision). Patients with concomitant liver and spleen injuries were assigned to either the liver or splenic injury group according to the organ with the higher injury grading [18]. High-grade BHSI are defined as grade III-VI blunt hepatic injuries and grade III-V blunt splenic injuries [18]. A total of 4379 patients were assessed for eligibility for the study. Among them, 4153 (94.8%) motorcyclists and 226 (5.2%) car occupants were enrolled in the study (Figure 1). Of these patients, 260 had sustained a BHSI, including 168 motorcyclists and 42 car occupants. Those patients who had sustained a low-grade injury (grade I or II), had no available abdomen computed tomography (CT) data, or who had incomplete registered data were excluded. Finally, 101 motorcyclists (2.4% of all motorcyclists) and 32 car occupants (14.1% of all car occupants) who had high-grade BHSI were eligible for further analysis.

### 2.3. Study Parameters

The data collected included age, sex, vital signs at the ED (systolic blood pressure (SBP), heart rate (HR), respiratory rate (RR), Glasgow Coma Scale (GCS) score, hemoglobin level, mechanism of injury, severity of hepatic and splenic injury, Injury Severity Score (ISS), Organ Injury Score (OIS), incidence of large hemoperitoneum or contrast pooling from CT, acute treatment procedures (tube thoracostomy, endotracheal intubation, blood transfusions), clinical management strategies (angiography, laparotomy), length of stay (LOS) in hospital and in intensive care unit (ICU), the presence of associated injuries, in-hospital mortality, and rates of associated complications.

### 2.4. Definitions

Unstable vital signs (defined as SBP ≤ 80 mmHg or RR > 20 times/min), large hemoperitoneum (defined as abnormal fluid accumulation over the subphrenic space, bilateral paracolic gutters, and pelvic cavity) or contrast pooling on CT, tube thoracostomy, blood transfusion >4 units (U), ISS > 16, and LOS in ICU >5 days were considered markers for the relative severity of injuries. ED blood transfusions included units of blood transfused during resuscitation at ED or transfusions before a transfer from a local hospital, whereas ward blood transfusions referred to all units administered during hospitalization, excluding those administered at the ED. Complications included intra-abdomen abscess, prolonged ileus, biloma, empyema, and a return to operating room for peritonitis, re-bleeding, or a failed conservative treatment.

### 2.5. Statistical Analysis

The data collected were compared using IBM SPSS Statistics for Windows, version 20.0 (IBM Corp., Armonk, NY, USA). Two-sided Fisher’s exact or Pearson’s chi-square tests were used to compare categorical data. The unpaired Student’s *t*-test was used to analyze normally distributed continuous data, which were reported as mean ± standard deviation. Mann–Whitney’s U test was used to compare non-normally distributed data. Moreover, *p*-values <0.05 were considered statistically significant.

## 3. Results

### 3.1. Characteristics of High-Grade BHSI in Motorcyclists and Car Occupants

In this study, the majority (76%, 101/133) of high-grade BHSI were due to motorcycle crashes. Among the 101 motorcyclists (2.4% of all motorcyclists) and 32 car occupants (14.1% of all car occupants) who had a high-grade BHSI, there was no statistically significant difference in sex and age between motorcyclists and car occupants (Table 1). In terms of vital signs on arrival at the ED, the SBP was significantly lower in car occupants than in motorcyclists (105 ± 28 vs. 122 ± 29 mmHg, respectively, *p* = 0.005), and RR was significantly higher in car occupants than in motorcyclists (22 ± 5 vs. 20 ± 4 times/min, respectively, *p* = 0.042). There were no significant differences in HR, hemoglobin level, or GCS score between the two groups. The car occupants had a significantly higher ISS (26.8 ± 10.9 vs. 20.7 ± 10.4, respectively, *p* = 0.005) and OIS (3.8 ± 1.0 vs. 3.4 ± 0.6, respectively, *p* = 0.033) than those in motorcyclists.

### 3.2. Management of Motorcyclists and Car Occupants with High-Grade BHSI

The units of blood transfused were significantly higher in the car occupants than in the motorcyclists (3.8 ± 4.9 vs. 2.0 ± 2.6 U, respectively, *p* = 0.048) admitted to the ED but not the ward. The percentage of performed acute management, including angiography and laparotomy at the ED or ward, was not significantly different between these two groups of patients. In addition, the hospital LOS was significantly longer for car occupants than for motorcyclists (21.2 days vs. 14.6 days, respectively, *p* = 0.038). However, although the stay in ICU was 3 days longer for car occupants than for motorcyclists, the difference was not statistically significant (8.1 days vs. 5.1 days, respectively, *p* = 0.065).

### 3.3. Clinical Presentation in Motorcyclists and Car Occupants with High-Grade BHSI

As shown in Table 2, car occupants with high-grade BHSI had worse clinical presentations than their motorcyclist counterparts, including a significantly higher incidence of hypotension (i.e., SBP ≤ 80), hyperpnea (i.e., RR > 20), severe injury (i.e., ISS > 16), tube thoracostomy, blood transfusion > 4 U, ICU LOS > 5 days, and complications. However, there were no differences in the incidence of contrast pooling or large hemoperitoneum on CT, endotracheal intubation, blood transfusion at the ED, or mortality between these two groups of patients.

### 3.4. Associated Injuries in the Thoracoabdominal Regions in Motorcyclists and Car Occupants with High-Grade BHSI

In the thoracoabdominal regions, there was a significantly lower incidence of clavicle fracture (0% vs. 14.9%, respectively, *p* = 0.021), but a higher incidence of chest injury (65.6% vs. 44.6%, respectively, *p* = 0.038) in car occupants than in motorcyclists (Table 3). There was no significant difference in the incidence of isolated rib fracture, hemothorax or pneumothorax, or lung contusion between these two groups of patients.

## 4. Discussion

In this study, the majority of BHSI were due to motorcycle crashes. This is different from the results reported in other countries, where car accidents have been found to account for the larger proportion of BHSI [2,3,6,19]. In addition, although motorcyclists are deemed to be unprotected and vulnerable to high-impact collisions, accounting for the high rates of morbidities and fatalities [9,10,12,17], car occupants were found to be at greater risk of high-grade BHSI than were motorcyclists, as there were 32 of 226 (14.1%) car occupants and 101 of 4153 (2.4%) motorcyclists had sustained high-grade BHSI in this study. The conflicting results between our study and those previously reported may be due to differences in the predominant mode of transportation and traffic regulations. In Taiwan, motorcyclists comprise a major portion of the trauma population and they are more likely to sustain injuries to the extremities [8,9,10,12,20,21], whereas thoracoabdominal injuries are more common in victims of car crashes [19,22,23,24]. Notably, although many studies have confirmed the effectiveness of safety belts in decreasing mortality rates and injury severity after motor vehicle crashes [8,25], seat belt use in the car is prone to specific regional injury patterns: patients whose records indicated the use of a seat belt had a 2.4-fold higher incidence of rib fractures, a 3.1-fold higher incidence of liver injuries, and a 24-fold higher incidence of splenic trauma compared to patients whose records did not indicate seat belt use [23].

In addition to carrying a greater risk for high-grade BHSI, with a significantly higher ISS and OIS, car occupants with high-grade BHSI were injured more severely than the motorcyclists. Moreover, car occupants had a higher incidence of worse clinical presentation, including hypotension, hyperpnea, tube thoracostomy, blood transfusion > 4 U, ICU LOS > 5 days, and complications. Notably, there were no significant differences in the incidence of either isolated rib fractures or hemothorax or pneumothorax, but there was a significantly higher incidence of tube thoracostomy in car occupants than in motorcyclists and more car crash victims (*n* = 11) received tube thoracostomy than those who had hemothorax or pneumothorax (*n* = 6). This suggests that the rib fracture was a more critical injury for car crash victims than for motorcyclists. Additionally, although the incidence of blood transfusion at the ED was similar between the two groups, the number of units of blood transfused at the ED was significantly higher for car occupants than for motorcyclists. Moreover, car crash patients had a significantly higher incidence of blood transfusion > 4 U during hospitalization than did motorcyclists. This is consistent with the results reported by Sartorelli et al. [26], in that clinical management is more critical in patients with BHSI who received more than 4 units of transfused blood, and the findings of Robinson III et al. [7], in that blood transfusion is a strong independent predictor of morbidity and hospital stay in patients with BHSI. However, it had to be noted that although in this study the car occupants with high-grade BHSI were injured more severely, had a higher incidence of worse clinical presentation and outcome than motorcyclists, it did not indicate the motorcyclists are safer than the car occupants regarding BHSI, because the comparison between motorcyclists and car occupants was not based on the same impact force during the accident but according to the selected patients with high-grade BHSI from the accident. The results rather indicated that since motorcyclists are not protected, it is easier to have an isolated BHSI; however, car occupants who are more protected would logically be more severely injured overall if the protection provided by a vehicle has failed to protect their spleen or liver. The results also implied that specific attention should be paid to those car occupants with high-grade BHSI, because there is an associated higher severity of injury and worse outcome of these patients. The critical condition of such patients should not be underestimated because of the concept that the patients within in a car are much safer.

After demonstration of the safety and effectiveness of non-operative management (NOM) of solid organ injuries [6,7,18,19,27,28], this protocol-based strategy has been accepted as a mainstream practice for select patients with BHSI. In the present study, the indicator for failure of conservative treatment is the rate of laparotomy required for patients during the stay in hospital, and this rate was not significantly different between motorcyclists and car occupants. The successful use of NOM for trauma patients with high-grade BHSI was demonstrated not only by the low incidence of NOM failure, but also by the non-significant difference in the overall mortality rate between motorcyclists and car occupants. The mortality rate was comparable to that reported in previous studies (10.5%–14.2%) [6,7,18,27]. However, taking into account that motorcyclists were approximately 30 times more likely to die in a motor vehicle crash than were motor vehicle occupants [29], and that motorcycle riders were 58 times more likely to be killed on a per-trip basis than their motor vehicle counterparts [30], the similar overall mortality between motorcyclists and car occupants in this study reflects the detrimental contribution of high-grade BHSI to mortality. Actually, isolated injuries have been reported to be uncommon in those sustaining high-grade BHSI [2,3,4,6,11,12,19,24,31]. Under the basic concept of a combination of injuries that cause a life-threatening condition [32,33], these polytrauma patients are expected to have a higher risk of morbidity and mortality than the summation of expected morbidity and mortality of their individual injuries [34]. In this study, most deaths were attributed to the associated injuries, including intracranial hemorrhage, pelvic fracture and coagulopathy, and multiple organ failure, but not to the high-grade BHSI per se. Therefore, management of concomitant injuries in patients with high-grade BHSI is important and should be given high priority [35].

Our study has some limitations that should be acknowledged. First, owing to the retrospective design of the study, there is inherent selection bias. Second, the patients declared dead on hospital arrival or at the accident scene were not included, and this may have led to selection bias. Third, the impact of pre-existing comorbidities on the course of hospitalization and mortality was not included in the analysis and, thus, remains unclear. Fourth, the lack of data regarding indication of hospitalization, acute management, and admission into ICU prevents further evaluation of the effects of any particular treatment intervention. This means that we could rely only on the assumption of uniform assessment and management of the studied populations.

## 5. Conclusions

This study demonstrated that car occupants with high-grade BHSI were injured more severely, had a higher incidence of worse clinical presentation, had a longer hospital LOS, and had a higher incidence of complications than motorcyclists. The results also implied that specific attention should be paid to those car occupants with high-grade BHSI, whose critical condition should not be underestimated because of the concept that the patients within in a car are much safer.

## Figures and Tables

**Table 1:** Characteristics and management of high-grade BHSI in motorcyclists and car occupants.

Variables	Motorcyclists	Car Occupants	*p*-Value
	*n* = 101	*n* = 32	
Sex (male)	64 (63.4%)	20 (62.5%)	0.929
Age (years)	32.3 ± 16.1	33.0 ± 15.4	0.843
SBP (mmHg)	122 ± 29	105 ± 28	**0.005**
HR (beats/min)	99 ± 21	100 ± 23	0.811
RR (times/min)	20 ± 4	22 ± 5	**0.042**
Hemoglobin (g/dL)	11.7 ± 2.5	11.7 ± 2.3	0.912
GCS	13.7 ± 2.8	13.3 ± 3.1	0.505
ISS	20.7 ± 10.4	26.8 ± 10.9	**0.005**
OIS	3.4 ± 0.6	3.8 ± 1.0	**0.033**
Blood transfusion (U), ED	2.0 ± 2.6	3.8 ± 4.9	**0.048**
Blood transfusion (U), ward	3.8 ± 6.8	6.3 ± 10.5	0.209
Angiography, ED	33 (32.7%)	8 (25.0%)	0.413
Angiography, ward	7 (6.9%)	4 (12.5%)	0.460
Laparotomy, ED	17 (16.8%)	9 (28.1%)	0.160
Laparotomy, ward	5 (5.0%)	1 (3.1%)	1.000
Hospital LOS (days)	14.6 ± 10.5	21.2 ± 16.1	**0.038**
ICU LOS (days)	5.1 ± 5.3	8.1 ± 8.4	0.065

ED: emergency department; GCS: Glasgow coma scale; HR: heart rate ICU: intensive care unit; ISS: injury severity score; LOS: length of stay; OIS: organ injury score; RR: respiratory rate; SBP: systolic blood pressure; U: unit. *p*-Values with statistical significance are presented in bold font.

**Table 2:** Clinical presentation in motorcyclists and car occupants with high-grade BHSI.

Variables	Motorcyclists		Car Occupants		*p*-Value
	*n*	%	*n*	%	
SBP ≤ 80 mmHg	9	8.9	8	25.0	**0.030**
RR > 20/min	20	19.8	12	37.5	**0.045**
ISS > 16	65	64.4	27	84.4	**0.033**
Contrast pooling in CT	31	30.7	9	28.1	0.783
Large hemoperitoneum in CT	31	30.7	11	34.4	0.696
Endotracheal intubation	13	12.9	4	12.5	1.000
Tube thoracostomy	17	16.8	11	34.4	**0.046**
Blood transfusion at ED	51	49.5	18	56.2	0.570
Blood transfusion > 4 U	30	29.7	16	50.0	**0.035**
ICU LOS > 5 days	28	27.7	16	50.0	**0.030**
Complications	7	6.9	7	21.9	**0.041**
Mortality	5	5.0	3	9.4	0.398

CT: computer tomography; ED: emergency department; GCS: Glasgow coma scale; ISS: injury severity score; ICU: intensive care unit; ISS: injury severity score; LOS: length of stay; RR: respiratory rate; SBP: systolic blood pressure; U: unit. *p*-Values with statistical significance are presented in bold font.

**Table 3:** Associated injuries in thoracoabdominal regions of motorcyclists and car occupants with high-grade BHSI.

Associated Injuries	Motorcyclists		Car Occupants		*p*-Value
	*n*	%	*n*	%	
Clavicle fracture	15	14.9	0	0	**0.021**
Scapula fracture	2	2.9	1	3.1	0.565
Chest injury	45	44.6	21	65.6	**0.038**
Isolated rib fracture	16	15.8	5	15.6	0.977
Hemothorax/pneumothorax	10	9.9	6	18.8	0.214
Lung contusion	5	5.0	0	0	0.336
Abdomen organ injury	20	19.8	7	21.9	0.799
Pelvic fracture	9	8.9	4	9.5	0.512

Abdominal organ injury included injury to the bowel, pancreas, kidney, and mesentery. *p*-Values with statistical significance are presented in bold font.
